# Linker-Free Magnetite-Decorated Gold Nanoparticles (Fe_3_O_4_-Au): Synthesis, Characterization, and Application for Electrochemical Detection of Arsenic (III)

**DOI:** 10.3390/s21030883

**Published:** 2021-01-28

**Authors:** Mohammed Sedki, Guo Zhao, Shengcun Ma, David Jassby, Ashok Mulchandani

**Affiliations:** 1 Materials Science and Engineering Program, University of California, Riverside, CA 92521, USA; mabue002@ucr.edu; 2 Department of Chemical and Environmental Engineering, University of California, Riverside, CA 92521, USA; zhaoguo@njau.edu.cn; 3 Department of Civil and Environmental Engineering, University of California, Los Angeles, CA 90095, USA; shengcun@ucla.edu (S.M.); jassby@ucla.edu (D.J.); 4 Center for Environmental Research and Technology (CE-CERT), University of California, Riverside, CA 92507, USA

**Keywords:** arsenic detection, linker-free, gold-magnetic nanoparticles, square wave anodic stripping voltammetry, ionic liquid

## Abstract

Linker-free magnetite nanoparticles (Fe_3_O_4_NPs)-decorated gold nanoparticles (AuNPs) were grown using a new protocol that can be used as a new platform for synthesis of other intact metal–metal oxide nanocomposites without the need for linkers. This minimizes the distance between the metal and metal oxide nanoparticles and ensures the optimum combined effects between the two material interfaces. X-ray diffraction (XRD) and Fourier transform infrared (FTIR) spectroscopy confirmed the successful synthesis of the Fe_3_O_4_-Au nanocomposite, without any change in the magnetite phase. Characterization, using transmission electron microscopy (TEM), scanning electron microscopy (SEM) and energy dispersive X-ray (EDX) spectroscopy, revealed the composite to consist of AuNPs of 70 ± 10 nm diameter decorated with tiny 10 ± 3 nm diameter Fe_3_O_4_NPs in Au:Fe mass ratio of 5:1. The prepared Fe_3_O_4_-Au nanocomposite was embedded in ionic liquid (IL) and applied for the modification of glassy carbon electrode (GCE) for the electrochemical detection of As(III) in water. By combining the excellent catalytic properties of the AuNPs with the high adsorption capacity of the tiny Fe_3_O_4_NPs towards As(III), as well as the good conductivity of IL, the Fe_3_O_4_-Au-IL nanocomposite showed excellent performance in the square wave anodic stripping voltammetry detection of As(III). Under the optimized conditions, a linear range of 1 to 100 μg/L was achieved with a detection limit of 0.22 μg/L (S/N = 3), and no interference from 100-fold higher concentrations of a wide variety of cations and anions found in water. A very low residual standard deviation of 1.16% confirmed the high precision/reproducibility of As(III) analysis and the reliability of the Fe_3_O_4_-Au-IL sensing interface. Finally, this proposed sensing interface was successfully applied to analyzing synthetic river and wastewater samples with a 95–101% recovery, demonstrating excellent accuracy, even in complex synthetic river and wastewater samples containing high concentrations of humic acid without any sample pretreatments.

## 1. Introduction

The contamination of arsenic in water is a significant concern to human health [1,2,3], as exposure can lead to a range of acute and chronic diseases, such as dysphasia, facial edema, dehydration, jaundice, and cancer [4,5,6,7,8,9,10]. Inorganic arsenic compounds, arsenite (As(III)) and arsenate (As(V)) commonly exist in the environment either due to geochemical enrichment or industrial processes. Arsenite and arsenate are more toxic than the organic forms. Arsenite, which is the most toxic, is a mobile and soluble form of arsenic [11]. Usually, the content of As(III) in water is a serious problem, because even though its concentration is very low, it is highly toxic even at trace levels and can enter the human body in different ways, causing serious health problems. The World Health Organization (WHO) has recommended an upper limit of 10 ppb for total arsenic in drinking water [11]. 

Different analytical strategies based on spectrometry, such as atomic fluorescence spectrometry [12], inductively coupled plasma mass spectrometry (ICP-MS) [13] and graphite furnace atomic absorption spectrometry [14] are frequently used to determine As(III) at trace levels in a variety of samples. While these techniques have high sensitivity for As(III) detection, they have limitations of high operating cost, expensive instruments, requirement of professional operators and bulky instrumentation, which make them unsuitable for on-site routine analysis.

In contrast to these methods, electrochemical analyses, and in particular, anodic stripping voltammetry (ASV), is a low cost, low limit of detection (LOD) sensing method that has the benefit of convenient operation, high sensitivity, and the ability to perform real-time analysis [15,16]. Usually, the fabrication of an electrochemical sensor based on electrode modification can be divided into the following steps [15,16,17,18]: 1. nanocomposites synthesis and characterization; 2. electrode fabrication and pretreatment; 3. electrode modification using the synthesized nanocomposites. Electrodes of various nano-materials/particles, such as carbon nanotubes, metal oxides, noble metals (Au, Ag and Pt), and graphene, have been used for As(III) determination [15,16,17,18]. Previous reports demonstrated that AuNPs-modified electrodes could enhance the anodic current response toward As(III) more effectively than other materials relying on chemical reduction by electrogenerated H_2_ [19,20,21,22,23,24,25]. However, these methods need strong acidic conditions to guarantee enough electrogenerated H_2_ availability for electrochemical reduction of As(III) to As(0) during the electrochemical reduction/deposition step. This limits the application of Au-based materials for the detection of As(III) by non-professionals and in the field.

Recently, a number of Fe_3_O_4_-based materials have been synthesized for arsenic removal because of the excellent arsenic adsorption ability of Fe_3_O_4_ [26,27]. In addition, some studies have been performed utilizing Fe_3_O_4_-based materials to analyze the concentration of As(III) [28,29]. However, the poor conductivity of Fe_3_O_4_ has limited its sensitivity for electrochemical detection. To improve the electrode conductivity and electrocatalytic activity, electrodes modified with Au-Fe_3_O_4_ nanocomposite were reported [30,31]. However, the synthesis procedures used linkers such as oleic acid, oleyl amine, or aminopropyl trimethoxy silane to attach/decorate Au nanoparticles on Fe_3_O_4_, required a high temperature and high-pressure autoclave for Fe_3_O_4_ synthesis, and were time-consuming (~24 h). The linker introduces separation between the adsorbed As(III) on magnetite and the Au nanoparticle catalyst that could potentially reduce electron transfer rate/efficiency [32]. Moreover, a large fraction of the adsorbing material surface area was covered/blocked by Au NPs.

Here, we report linker-free grown AuNPs decorated with Fe_3_O_4_NPs (Fe_3_O_4_-Au) nanocomposite using a facile, faster and green chemistry synthesis route for application in chemically modified electrode ASV detection of As (III) in water. The important differences in the nanocomposite material between this work and the literature include the morphology/architecture and synthesis method as follows. (1) There is no linker between Fe_3_O_4_ and Au, which minimizes the distance between the metal and metal oxide nanoparticles and ensures the optimum combined effects between the two material interfaces. (2) Nanocomposite consists of AuNPs (70 nm average diameter) decorated with very tiny (10 nm average diameter) Fe_3_O_4_NPs which provide a higher surface area of the adsorbent for As(III). (3) The synthesis uses a co-precipitation method that is performed at low temperature (80 °C) and atmospheric pressure and does not require harsh organic solvents to synthesize Fe_3_O_4_NPs. 

The introduced material was used to modify the surface of a glassy carbon electrode (Fe_3_O_4_-Au-IL/GCE) and applied as a working electrode in the ASV detection of As(III). The results exhibited a comparable or higher sensitivity than the modified electrodes reported in the literature. The combination of AuNPs, Fe_3_O_4_ NPs, and IL resulted in many intriguing combined effects and catalytic abilities, such as an excellent ability to adsorb As(III), high electrical conductivity and good stability. In addition, the synthesis method introduced in this work can be used as a new platform for synthesis of intact metal-Fe_3_O_4_ nanocomposites without the need for any linkers to achieve the optimum combined effects between the materials. Moreover, the electrode materials were characterized using different techniques (CV, SEM, EDS, XRD, TEM and EIS). Furthermore, a dual-catalysis system has been proposed for the first time to investigate and explain the catalytic mechanism behind the phenomenon. Finally, the analytical application of Fe_3_O_4_-Au-IL/GCE was tested for the measurement of As(III) in synthetic river water and wastewater samples.

## 2. Experimental

### 2.1. Reagents and Instruments

Ferrous chloride (FeCl_2_·4H_2_O), ferric chloride (FeCl_3_·6H_2_O), ethylene glycol (EG), ammonium hydroxide (NH_4_OH, 25%), sodium citrate, sodium hydroxide (NaOH), hydrogen tetrachloroaurate (HAuCl_4_·3H_2_O), polyvinyl alcohol (PVA), sodium acetate (CH_3_COONa), ammonium acetate (CH_3_COONH_4_), monopotassium phosphate (KH_2_PO_4_), sodium bicarbonate (NaHCO_3_), manganese sulfate (MnSO_4_), zinc sulfate (ZnSO_4_), magnesium sulfate (MgSO_4_), and calcium chloride (CaCl_2_) were purchased from Fisher Scientific (USA). Humic acid sodium salt (Technical grade) was purchased from Sigma-Aldrich. Ionic liquid ([C4dmim][NTf_2_]) was obtained from IoLiTec Ionic Liquids Technologies, INC. (USA). Arsenic trioxide (As_2_O_3_) was purchased from Strem Chemicals, INC. (USA). An As(III) stock solution (1 mg/mL) was prepared by dissolving As_2_O_3_ in the 1.0 M aqueous NaOH. Sodium acetate trihydrate was obtained from Fisher Scientific (USA) and prepared as acetate buffer solution (0.2 M) with acetic acid for the electroanalysis of As(III).

The prepared Fe_3_O_4_NPs (magnetite), AuNPs, and Fe_3_O_4_-Au were characterized by scanning electron microscopy (SEM, Zeiss 1540 XB Crossbeam scanning electron microscope.), transmission electron microscopy (TEM, FEI Tecnai12), energy dispersive X-ray spectroscopy (EDX), X-ray diffraction (XRD, PANalytical Empyrean Series 2), and Fourier transform infra-red (FTIR, Thermo Nicolet 6700) spectroscopy. Electrochemical analysis, i.e., cyclic voltammetry (CV), square wave anodic stripping voltammetry (SWASV), and electrochemical impedance spectroscopy (EIS) were performed on a CH Instrument 760C electrochemical workstation. A three-electrode system and a 20 mL cell were used for all measurements, in which Fe_3_O_4_-Au-IL/GCE, Pt wire, and Ag/AgCl electrode were used as working, counter, and reference electrodes, respectively. During the deposition step, the test solution was stirred using a magnetic stir bar. The As(III) in synthetic wastewater detected by ICP-MS (PerkinElmer NexION 2000) was used to confirm the robustness of our synthesis method in complex water streams. 

### 2.2. Synthesis and Modification of Fe_3_O_4_-Au-IL Nanocomposite

In this procedure, Fe_3_O_4_NPs were prepared using the co-precipitation method, as reported earlier [33], with some modifications. The de-ionized (DI) water and reagents used in this procedure were all de-oxygenated before use. In brief, a mixture of FeCl_2_·4H_2_O and FeCl_3_·6H_2_O (20 mg and 32 mg, respectively) was added to the solution consisting of 20 mL of DI water and 20 mL of EG. The mixed solution was heated to 60 °C with stirring under nitrogen purging. Ammonia solution (5%) was added dropwise to the above solution until reaching the pH of 10, and then the reaction mixture was agitated for half an hour. The obtained mixture was washed a few times with DI water by means of an external magnet to remove the excess ammonia solution and surfactant. Quantities of 10 mL of HAuCl_4_·3H_2_O (20 mg/mL) and 10 mL of EG were added to the obtained Fe_3_O_4_NPs and stirred for 2 min at 80 °C. Then, 5 mL of sodium citrate (0.3%) was added to the Fe_3_O_4_-Au^3+^ solution and stirred at 80 °C for half an hour to obtain the Fe_3_O_4_-Au nanocomposite. The prepared suspension was washed with DI water by means of an external magnet to remove the excess surfactants and the free AuNPs that were not bonded to magnetite, if any. A quantity of 4 mL of Fe_3_O_4_-Au nanocomposite solution (1 mg/mL) was mixed with 300 μL of 0.5% ionic liquid (IL) in ethanol to obtain the Fe_3_O_4_-Au-IL hybrid structure. The surface of GCE was polished by 0.05 μm alumina powder before modification, and was then immersed in 1:1 HNO_3_, absolute ethanol and water for sonication, separately. Subsequently, 8 μL of the Fe_3_O_4_-Au-IL suspension was drop-casted onto the surface of GCE and dried in oven at 60 °C. Other electrodes were prepared the same way as described above.

### 2.3. Stripping Voltammetry Analysis of As(III)

The electrochemical measurements of SWASV were performed in a 0.2 M acetate buffer solution (pH 5.0) containing different concentrations of As(III). Unless stated otherwise, −0.9 V was used as a deposition/reduction potential for the pre-deposition of As(III) under stirring for 200 s. The stirring was stopped once the deposition step was completed. An anodic stripping voltammogram was obtained from −0.4 to 0.3 V after an equilibration period of 10 s. The frequency, amplitude and potential step were 25 Hz, 25 mV and 5 mV, respectively. To regenerate the modified electrode before the next measurement at the end of stripping, an oxidation potential of 0.6 V was applied to the working electrode for 120 s to remove any As(0) residuals which may not be completely stripped from the electrode surface.

### 2.4. Preparation of Water Samples

DI water containing 150 ppm magnesium nitrate, 60 ppm ammonia chloride, 500 ppm calcium chloride, 50 ppm sodium citrate and 500 ppm potassium calcium chloride (1260 ppm total dissolved solids), simulating the chemical composition of Yamuna River in Northern India, was spiked with As(III) and tested with the developed sensor to evaluate sensor performance. To further validate the robustness of our electrode, the electrode was used to measure As(III) in synthetic wastewater, the recipe of which was modified from the literature [34] and listed in Table S1. In these experiments, a 10 mL water sample test solution composed of 9 mL simulated water and 1 mL 2 M acetate buffer (pH 5.0) was used for each measurement to ensure the pH 5.0 buffer condition with 0.2 M acetate.

## 3. Results and Discussion

In this work, we employ the concept of adsorbent-assisted stripping voltammetry analysis for sensitive detection of As(III) based on a sensing interface of AuNPs decorated with Fe_3_O_4_NPs (Fe_3_O_4_-Au). AuNPs have excellent catalytic properties and the Fe_3_O_4_NPs are highly adsorptive to As (III), which makes Fe_3_O_4_-Au a very promising material for the stripping voltammetry analysis of As(III). Additionally, ionic liquids (IL) have high adhesiveness, conductivity, work across a wide potential window, and have been widely used in the modification of electrodes [35,36,37,38,39,40]. Incorporating IL into the nanocomposite should further enhance the sensitivity and stability of the sensing interface on the electrode surface. 

**Figure 1:**
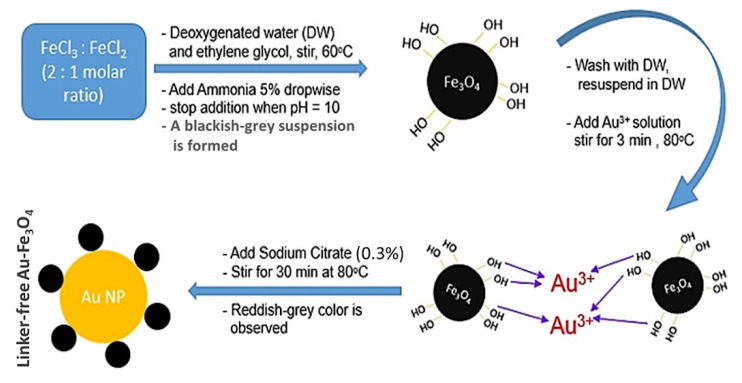
Schematic of Fe_3_O_4_-Au nanoparticle preparation and the suggested mechanism of its formation.

The synthesis method introduced in this work can be used as a new platform for synthesis of intact metal-Fe_3_O_4_ nanocomposites without the need for any linkers, which minimizes the distance between the metal and metal oxide nanoparticles and ensures the optimum combined effects between the materials. In this method, EG could provide magnetite with hydroxyl groups that facilitate the chelation of Au^3+^ ions (Fe_3_O_4_-Au^3+^), as illustrated in the schematic diagram of Figure 1. In this novel platform method, sodium citrate is a mild reducing and capping agent that can reduce gold ions slowly into AuNPs [41,42], while it will not reduce Fe^3+/2+^ in magnetite and hence it enables the synthesis of the Fe_3_O_4_-Au nanocomposite in situ without any linkers. 

### 3.1. Physicochemical Characterization of Fe_3_O_4_-Au Nanocomposite

**Figure 2:**
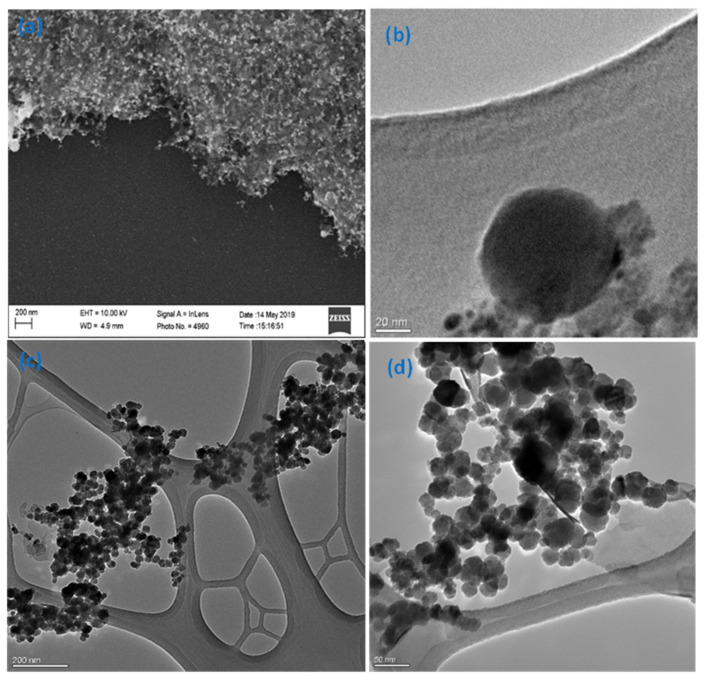
Electron microscopy images. (**a**) SEM image of a thin film of Fe_3_O_4_-Au nanocomposite on a silicon wafer sputtered with Pd/Pt, (**b**) a higher resolution TEM image of the prepared nanocomposite, and (**c**,**d**) TEM images of the Fe_3_O_4_NPs.

The introduced synthesis method is time saving (1–2 h), as the synthesis of magnetite NPs was conducted using the co-precipitation method. It is also linker-free with no barrier between gold and magnetite, which improves the electron transfer between them and enhances their combined effects. This procedure uses simple chemicals that are mostly ecofriendly, and very easy to wash using deionized water, if any excess is present. In addition, magnetite nanoparticles were prepared to be very tiny (≈10 nm) to allow for higher surface area and higher adsorption of As(III). The morphology and sizes of the prepared particles were assessed using SEM and TEM imaging as illustrated in Figure 2. The synthesized Fe_3_O_4_-Au suspension was drop-casted as a thin film on a piece of silicon wafer, at the same concentration and volume used in preparing the electrode materials, and sputtered with Pd/Pt to reduce surface charging, and then imaged by SEM. The SEM image in Figure 2a illustrates that AuNPs (the bigger and brighter particles) are surrounded by much smaller Fe_3_O_4_NPs. However, the Fe_3_O_4_NPs were not well resolved. Further imaging of Fe_3_O_4_-Au by TEM (Figure 2b) shows a 70 nm spherical core AuNP decorated with Fe_3_O_4_NPs, which agrees with the SEM image. The TEM image of Fe_3_O_4_NPs showed the particles are spherical with an average size of 10 ± 3 nm (Figure 2c,d). The TEM images confirm the success of the proposed platform, as the AuNPs and Fe_3_O_4_NPs are directly bound to each other without separation between them. Furthermore, EDX spectroscopy was implemented to calculate the actual weight ratio between Au and Fe in the prepared nanocomposite. The results in Supplementary Figure S1, determined that the weight ratio is approximately 5:1 (Au:Fe).

**Figure 3:**
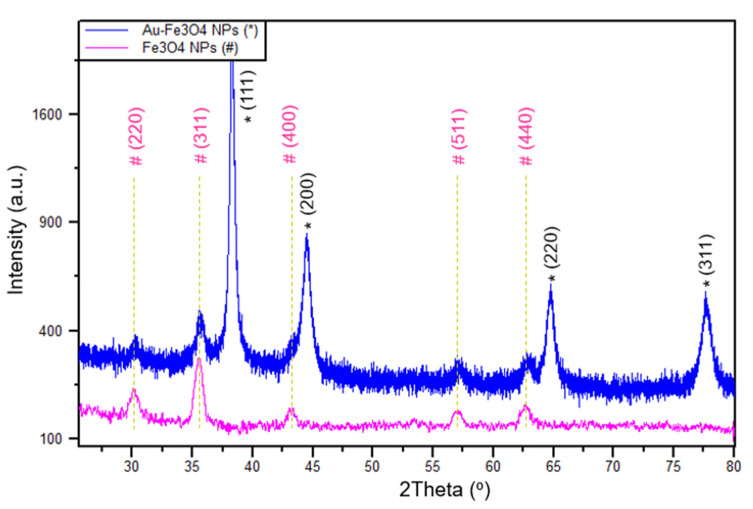
XRD patterns of Fe_3_O_4_NPs and Fe_3_O_4_-Au.

**Figure 4:**
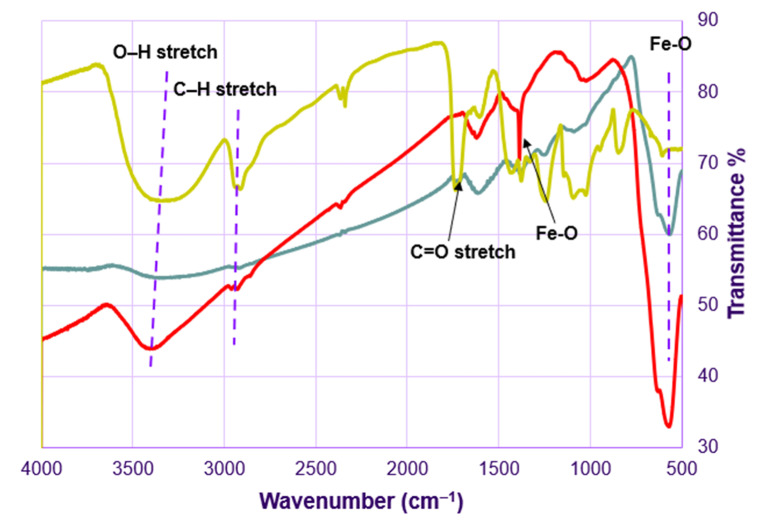
FTIR spectra of Fe_3_O_4_ (red), Au (yellow), and Fe_3_O_4_-Au (blue).

The results of the XRD analysis of magnetite (Fe_3_O_4_) NPs in Figure 3 show diffraction peaks at 30.17°, 35.53°, 43.27°, 56.96°, 62.69°, which can be assigned to the (220), (311), (400), (511), and (440) planes of the cubic inverse spinel-type structure of magnetite (PDF#85-1436) [43,44]. Moreover, the diffraction pattern of Fe_3_O_4_-Au has the same peaks of magnetite addressed above in addition to the characteristic peaks of the face-centered cubic (FCC) AuNPs at 38.23°, 44.54°, 64.71°, 77.69°, which can be assigned to the (111), (200), (220), and (311) planes, (Pattern 4-784) [45]. Hence, the XRD data confirm the successful synthesis of the Fe_3_O_4_-Au nanocomposite, without any change in the magnetite phase. Moreover, the intensity of AuNPs peaks is higher because they are in a higher concentration, which agrees with the EDS data, in which the weight ratio is around 5:1 (Au:Fe) (Figure S1). FTIR spectrum analysis (Figure 4) of Fe_3_O_4_NPs shows a broad peak centering at 3380 cm^−1^ and two peaks at 2900 and 2915 cm^−1^, which are attributed to the stretching vibrations of O-H and C-H groups from EG, respectively. In addition, Fe-O vibrations show two peaks at 1380 and 565 cm^−1^. Vibrational peaks of Fe_3_O_4_NPs are in a good agreement with published work [46]. On the other hand, the spectrum of AuNPs shows stretching vibrations of O-H groups of PVA at 3320 cm^−1^, vibrations of C=O groups of sodium citrate at 1730 cm^−1^ and stretching vibrations of C-H groups at 2900 and 2915 cm^−1^. The FTIR spectrum of Fe_3_O_4_-Au contains all the peaks from both spectra of Au and Fe_3_O_4_, which confirms the successful synthesis of this nanocomposite. 

### 3.2. Electrochemical Characterizations of Modified Electrodes

The electrochemical characteristics of bare GCE and different modified GCEs, i.e., Fe_3_O_4_NPs/GCE, Fe_3_O_4_-Au/GCE, and Fe_3_O_4_-Au-IL/GCE were investigated using CV based on a ferri/ferrocyanide [Fe(CN)_6_]^3−/4−^ redox probe (Figure S2A). On the bare GCE, two well-defined redox peaks of [Fe(CN)_6_]^3−/4−^ were found in curve a. When compared to the bare GCE, Fe_3_O_4_NPs/GCE showed a decreased redox current of [Fe(CN)_6_]^3−/4−^, because of the poor conductivity of Fe_3_O_4_. However, stronger redox peaks were found with the Fe_3_O_4_-Au/GCE (curve c) compared to the Fe_3_O_4_NPs/GCE, due to the excellent electrical conductivity of the AuNPs. Furthermore, the addition of IL to the nanocomposite, i.e., Fe_3_O_4_-Au-IL/GCE, increased its conductivity and resulted in stronger redox peaks. 

EIS was used to explore the material/electrode interface properties and changes based on the impedance changes (Figure S2B). The EIS results show different semicircles, each with a diameter equivalent to the charge transfer resistance (R_ct_), which corresponds to the process of electron transfer limitation [47,48,49]. Based on the value of semicircle diameter, the order of the R_ct_ of different modified electrodes was as follows: Fe_3_O_4_/GCE (curve b) > Fe_3_O_4_-Au/GCE (curve c) > Fe_3_O_4_-Au-IL/GCE > bare GCE (curve a). The EIS results are consistent with those of CV.

### 3.3. Optimization of Analysis Parameters

**Figure 5:**
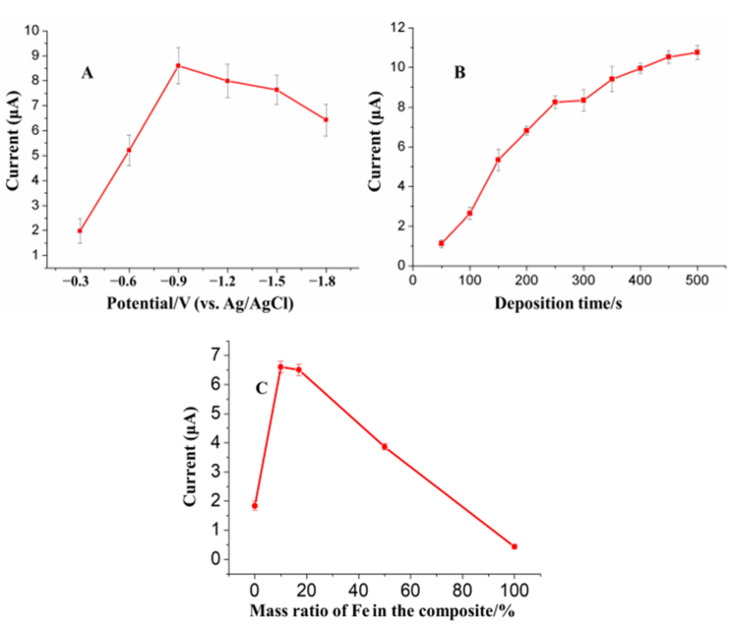
(**A**) Effect of the deposition potential, (**B**) deposition time and (**C**) weight ratio between Fe and Au on stripping current for 20 ppb (**A**,**B**) and 50 ppb (**C**) of As(III) in 0.2 M pH 5.5 acetate buffer at Fe_3_O_4_-Au-IL/GCE. Each data point is an average of 5 measurements from 3 electrodes and error bars represent ±1 standard deviation.

Deposition potential and deposition time play a key role in the SWASV sensitivity (stripping peak current/concentration) of heavy metals detection. Additionally, Fe_3_O_4_NPs and AuNPs play a unique role in the dual-catalytic system for the SWASV detection of As(III). Therefore, in this study, responses of Fe_3_O_4_-Au-IL/GCE toward As(III) were investigated to find optimal values of deposition potential, deposition time and the mass ratio of Fe_3_O_4_ to AuNPs. Figure 5A shows the influence of deposition potential on the stripping peak current of 20 ppb As(III) for 200 s deposition time and Fe:Au of 1:5. As shown in the figure, the stripping peak current increased with an increase of deposition potential reaching a maximum at −0.9 V followed by a small decrease at deposition potential above −0.9 V. The lower currents at potentials below −0.9 V are ascribed to incomplete reduction of As(III). On the other hand, the small decrease in current at deposition potentials above −0.9 V are the result of hydrogen evolution. Thus, the deposition potential of −0.9 V was chosen for As(III) preconcentration. 

The effect of deposition time on the peak current of 20 ppb As(III) at −0.9 V deposition potential and Fe:Au of 1:5 is shown in the Figure 5B. The results show a monotonic increase in peak current as a function of deposition time. The direct relation between the current and deposition time suggests a lower LOD could be expected by increasing the deposition time. However, as a longer deposition time implies a longer analysis time, we selected a 200 s deposition period. 

Different mass ratios of AuNPs to Fe_3_O_4_NPs in Fe_3_O_4_-Au composite on the stripping peak current of 50 ppb As(III) at −0.9 V deposition potential and 200 s deposition time were examined (Figure 5C). The results show an increase in the stripping peak current from 1.8 μA to ~6.6 μA when the Fe_3_O_4_-Au composite contained 10 to 16.3% (by mass) of Fe followed by a rapid drop to ~0.3 μA for 100% Fe (i.e., Fe_3_O_4_NPs only). This indicates a clear complementarity relationship between the two components of the nanocomposite in enhancing the sensitivity of the sensor for As(III) detection. It is interesting that although Fe_3_O_4_NPs have a strong adsorption capacity for the As(III), the response on Fe_3_O_4_NPs/GCE was the smallest. That may be ascribed to the poor conductivity of Fe_3_O_4_NPs, which affects both reduction of As(III) to As(0) and the oxidation of As(0) to As(III) on the electrode surface. Additionally, while the stripping response on AuNPs/GCE was higher than Fe_3_O_4_NPs/GCE, it was lower than Fe_3_O_4_-Au/GCE at different Fe mass ratios. This may be attributed to the combined effect of the strong adsorption capacity of Fe_3_O_4_ and good conductivity and catalytic ability of AuNPs. Based on cost and maximum sensitivity, Fe_3_O_4_-Au nanocomposite containing 16.3% (by mass) Fe was selected as the optimum composition.

### 3.4. Analytical Characteristics of Fe_3_O_4_-Au-IL/GCE for As(III)

**Figure 6:**
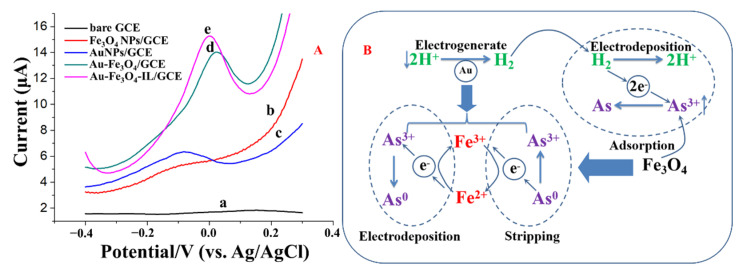
(**A**) The SWASV responses of 50 μg/L As(III) on different modified electrodes; (a) bare GCE, (b) Fe_3_O_4_NPs/GCE, (c) AuNPs/GCE, (d) Fe_3_O_4_-Au/GCE and (e) Fe_3_O_4_-Au-IL/GCE. (**B**) The catalytic mechanism of Fe_3_O_4-_Au in the electroanalysis of As(III). Glassy carbon electrode (GCE); ionic liquid (IL).

The stripping responses of 50 μg/L As(III) on bare GCE and different modified electrodes are shown in Figure 6. As shown in Figure 6A, almost no stripping response was obtained on the bare GCE. The stripping response of As(III) on Fe_3_O_4_NPs/GCE was not significant either, due to the poor conductivity of Fe_3_O_4_NPs. In contrast, at the Fe_3_O_4_-Au/GCE, a higher stripping peak signal was obtained due to the combined effect of Fe_3_O_4_NPs and AuNPs, which can be ascribed to the dual-catalysis system (Figure 6B). H_2_ was electrogenerated on the AuNP decorated with Fe_3_O_4_NPs at the applied negative potential during the electrodeposition step. The generated H_2_ then donates electrons in the reduction of As(III) to As(0), and forms H^+^ ions. Due to the strong adsorption capacity of Fe_3_O_4_NPs, As(III) concentration near the electrode surface gradually increased, which enhanced the catalytic efficiency of H_2_ on the As(III) adsorbed to Fe_3_O_4_NPs even at a high pH condition (the first catalytic system). Furthermore, the surface-activated Fe(II) can also donate an electron to form Fe(III), which helps reduce As(III). The produced Fe(III) will then acquire an electron, to revert to Fe(II), from either the electrode or the oxidation of As(0) to As(III) during the stripping process of SWASV. The procedure described above is a complete Fe(II)/Fe(III) cycle. During this process, the electron transfer between electrode and As(III) was mediated by Fe(II), which was used as an electrocatalyst (the second catalytic system). This mediation of the Fe(II)/Fe(III) cycle together with the catalysis of AuNPs will efficiently promote the deposition of As(III) and further enhance the sensitivity toward the electrochemical detection of As(III) [23,50]. Additionally, the stripping peak current was further improved with Fe_3_O_4_-Au-IL/GCE due to the good conductivity and adhesiveness of IL.

#### 3.4.1. Sensitivity, Limit of Detection, and Reproducibility

**Figure 7:**
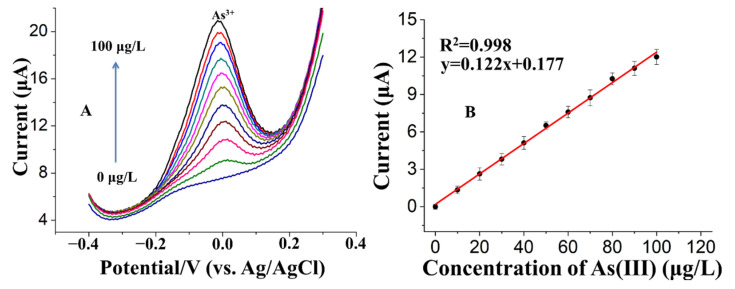
(**A**) SWAS voltammograms for additions of 0, 10, 20, 30, 40, 50, 60, 70, 80, 90 and 100 μg/L As(III). (**B**) The corresponding calibration curve of As(III).

The Fe_3_O_4_-Au-IL/GCE operating at optimal Fe:Au mass ratio, deposition potential, and deposition time determined above, was applied to analyze 0 to 100 ppb of As (III) in 0.2 M pH 5 acetate buffer (Figure 7). The results show well-defined Gaussian shaped response with peak current around 0 V which increased with increasing As(III) concentration (Figure 7A). As illustrated in Figure 7B, the peak stripping current, i.e., response, was linearly related to As(III) concentration over the complete concentration range of 0 to 100 ppb. The sensitivity (the slope of the calibration plot) was 0.122 mA/ppb As(III) and the LOD (calculated based on S/N = 3) was 0.22 ppb. The high sensitivity and low LOD is credited to the combined effects of excellent absorption ability for As(III), high electrical conductivity and good stability of AuNPs_,_ Fe_3_O_4_ NPs and IL. Table S2 shows a comparison of this work to the previous literature in terms of electrode material, technique, linear range, and limit of detection.

Furthermore, the developed sensor demonstrated excellent reproducibility as evidenced by a very low (1.16%) relative standard deviation (RSD) for five repetitive measurements of 60 ppb As(III) (Figure S3).

#### 3.4.2. Selectivity

**Figure 8:**
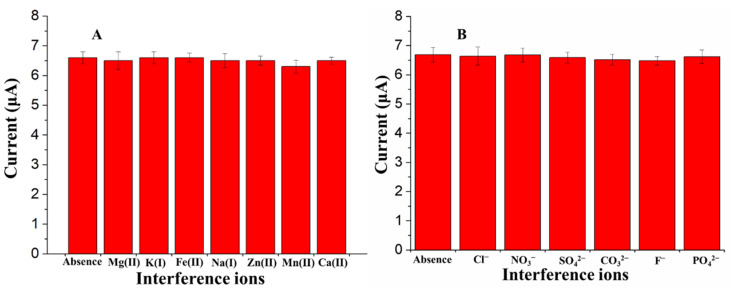
The selectivity of the proposed sensor in the presence of high concentrations of different ions (**A**: Cation; **B**: Anion) on the stripping peak current of 50 μg/L As(III) at Fe_3_O_4_-Au-IL/GCE in 0.2 M acetate buffer (pH 5.0). Each data point is an average of 5 measurements from 3 electrodes and error bars represent ±1 standard deviation.

Water sources contain a plethora of anions and cations that can potentially have a negative influence on the SWASV detection accuracy of As(III). We investigated the selectivity of the proposed sensor based on the analysis of 50 ppb of As(III) from 100-fold higher concentrations of Na^+^, K^+^, Ca^2+^, Zn^2+^, Mg^2+^, Mn^2+^ and Fe^2+^, and a 150-fold higher concentrations of Cl^−^, NO_3_^−^, SO_4_^2−^, CO_3_^2−^, F^−^ and PO_4_^2−^. As shown in Figure 8, there were no measurable changes in the peak current signals (current variation < ± 10%) in the presence of all the tested ions, confirming the proposed Fe_3_O_4_-Au-IL/GCE is efficient in the detection of As(III) in the presence of non-target species in water.

### 3.5. Analysis of Simulated Water Samples

To evaluate the applicability of Fe_3_O_4_-Au-IL/GCE for practical application, the sensor was applied to detect 5, 10 and 15 ppb of As(III) spiked in simulated river water representing the chemical composition of Yamuna River water in Northern India. Yamuna River is one of the most polluted rivers in India, and perhaps the world, with a complex matrix of pollutants [51,52]. As shown in Table 1, the recoveries (i.e., agreement) ranged from 97.2% to 101.3% (average of 99.06%) and the RSD ranged from 3.3% to 4.8% (average 3.9%). The excellent recoveries and low RSD demonstrate the promise of the developed sensor for monitoring As(III) in real environmental samples. To further validate Fe_3_O_4_-Au-IL/GCE’s robustness in complex wastewater streams, we used the sensor to detect As(III) in synthetic wastewater samples containing high concentrations of humic acid (50 mg/L), and compared it with values measured using ICP-MS. The Fe_3_O_4_-Au-IL/GCE sensor successfully determined As(III) concentrations, which were comparable to the results generated by ICP-MS (i.e., 95.4% ±4.7%) indicating that Fe_3_O_4_-Au-IL/GCE is a robust electrode for detecting As(III) in complex water streams.

## 4. Conclusions

In this work, an effective sensitive interface was designed using AuNPs decorated with Fe_3_O_4_NPs embedded in IL for the determination of As(III) at a trace level based on adsorbent-assisted in-situ electrocatalysis. The procedure for Fe_3_O_4_-Au synthesis introduced in this work (1) produced very tiny (≈10 nm) Fe_3_O_4_NPs enhancing the surface area for As(III) adsorption, (2) did not use a linker between gold and magnetite, which enhanced their combined effects, (3) is a green chemistry approach and (4) greatly reduced synthesis time (1–2 h). The proposed Fe_3_O_4_-Au-IL/GCE significantly enhanced the stripping response of As(III) as compared with the Fe_3_O_4_/GCE and AuNPs/GCE, as it provided a specific interface for As(III) to transfer electrons. Additionally, a Fe(II)/Fe(III) cycle and electrogenerated H_2_ were activated to catalyze the reduction/stripping of the As(III)/As(0), which enabled a high sensitivity. The presence of IL in the composite contributed to better sensitivity and stability. The analytical, spectroscopic and microscopic features of the proposed Fe_3_O_4_-Au-IL/GCE characterization by XRD, EDX, TEM, SEM, EIS SWASV, and CV showed that the combined effect of AuNPs, Fe_3_O_4_ and IL resulted in a much better electrical conductivity, larger specific surface area and higher catalytic ability than the bare electrode. Moreover, several synthetic water samples were tested to further verify the practicability of the proposed platform. The results showed that Fe_3_O_4_-Au-IL/GCE can be successfully applied to the analysis of As(III) in synthetic river and wastewater samples without any sample pretreatment procedures, which suggested that it could be further applied to heavy metal analysis in real samples.

## Figures and Tables

**Table 1:** Detection of As(III) in simulated river water.

Sample No.	Found ^a^ (μg/L)	Added (μg/L)	Detected after Adding ^a^ (μg/L)	Mean Recovery (%)	RSD (%)
1	3.12 ± 1.08	5	7.98 ± 1.23	97.2	4.8
2	4.89 ± 0.89	10	15.02 ± 1.15	101.3	3.3
3	9.95 ± 0.96	15	24.75 ± 0.90	98.67	3.6

^a^ Mean value ± Standard Deviation. Each data point is an average ±1 standard deviation from 3 measurements using 3 electrodes.

## Data Availability

Data sharing is not applicable to this article.

## Supplementary Materials

The following are available online at https://www.mdpi.com/1424-8220/21/3/883/s1, Figure S1: EDX spectrum of the Fe_3_O_4_-Au nanocomposite, Figure S2: (A) Cyclic voltammograms and (B) Nyquist plot of electrochemical impedance spectra, Figure S3: Five repetitive stripping current measurements of 60 μg/L As(III) using the Fe_3_O_4_-Au-IL/GCE in 0.2 M acetate buffer, Table S1: The composition of synthetic raw wastewater, Table S2: Comparison of different electrodes for the detection of As(III).
