# Supplementary material for: Linker-Free Magnetite-Decorated Gold Nanoparticles (Fe3O4-Au): Synthesis, Characterization, and Application for Electrochemical Detection of Arsenic (III)
Source: Sensors (Basel). 2021 Jan 28;21(3):883. doi: 10.3390/s21030883 (PMC7866134; doi:10.3390/s21030883)
Supplement: Supplementary file 1 [file sensors-21-00883-s001.pdf]

Supplementary Information

Linker-Free Magnetite-Decorated Gold Nanoparticles (Fe_3O_4-Au): Synthesis, Characterization, and Application for Electrochemical Detection of Arsenic (III)

Mohammed Sedki [1,†], Guo Zhao [2,†,‡], Shengcun Ma [3], David Jassby [3] and Ashok Mulchandani [2,4,*]

[1] Materials Science and Engineering Program, University of California, Riverside, CA 92521, USA; mabue002@ucr.edu
[2] Department of Chemical and Environmental Engineering, University of California, Riverside, CA 92521, USA; zhaoguo@njau.edu.cn
[3] Department of Civil and Environmental Engineering, University of California, Los Angeles, CA 90095, USA; shengcun@ucla.edu (S.M.); jassby@ucla.edu (D.J.)
[4] Center for Environmental Research and Technology (CE-CERT), University of California, Riverside, CA 92507, USA
* Correspondence: adani@engr.ucr.edu
† These authors contributed equally to this work.
‡ Current address: College of Artificial Intelligence, Nanjing Agricultural University, Nanjing 210031, China

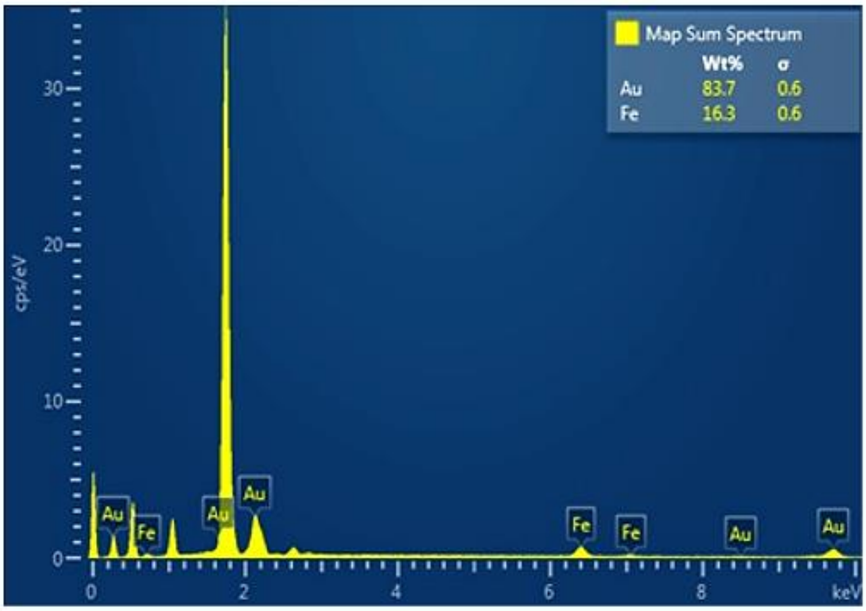

Figure S1. EDX spectrum of the Fe_3O_4-Au nanocomposite on a piece of Pd/Pt-sputtered silicon wafer with the weight percentage.

Table S1. The recipe of synthetic raw wastewater.

Compounds	Amount (mg) added to 500 mL
CH_3COONa	128.20
CH_3COONH_4	120.44
KH_2PO_4	21.97
$NaHCO_3$	62.50
$FeCl_2$	0.188
$MnSO_4$	0.02
$ZnSO_4$	0.18
NaCl	292.50
Humic acid sodium	25.00
$MgSO_4$	12.50

$CaCl_2$	5.00

Table S2. Comparison of different electrodes for the detection of As(III).

Electrodes	Technique	Linear range (μg/L)	LOD (μg/L)	Reference
AuNP/BDD electrode	SWASV	100-1500	20	[1]
Au-Pd/GCE	ASV	1-25	0.5	[2]
nano-Au/PANI/GCE	SWV	610-3050	0.4	[3]
AuNPs-PCWEs	CS	2-50	2.2	[4]
rGO/Fe_3O_4/SPCE	SWASV	2-20	0.3	[5]
AuNPs/GCE	SWASV	0-15	0.25	[6]
NanoPt-Fe(III)/MWCNT/GCE	ASV	1.35-14.5	0.75	[7]
Fe_3O_4-Au-IL/GCE	SWASV	0-100	0.22	This work

AuNP/BDD: gold nanoparticles on a boron-doped diamond; nano-Au/PANI/GCE: nanogold-particle/polyaniline-modified Glassy Carbon Electrode; SWV: Square Wave Voltammetric; AuNPs-PCWEs: gold-modified paper-based carbon working electrodes; CS: Chronoamperometric Stripping; rGO: reduced graphene oxide; SPCE: screen-printed carbon electrode; platinum-iron(III) nanoparticles modified multi-walled carbon nanotube on glassy carbon electrode: nanoPt-Fe(III)/MWCNT/GCE; GCE: glassy carbon electrode

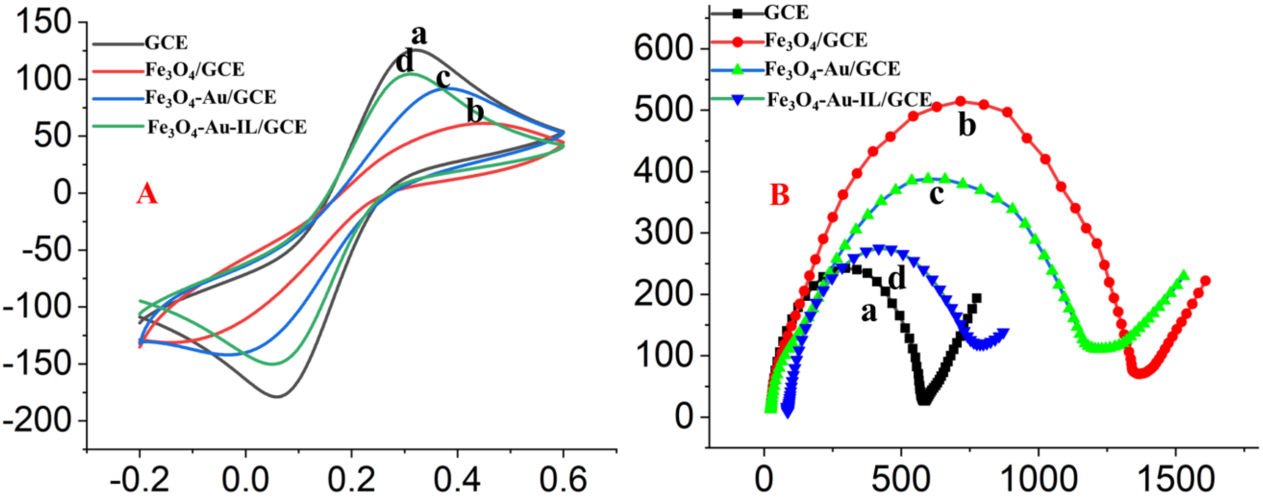

Figure S2. (A) Cyclic voltammograms and (B) Nyquist plot of electrochemical impedance spectra of 5mM $[Fe(CN)_6]^{3-/4-}$ in 0.1 M KCl: (a) bare GCE, (b) Fe_3O_4/GCE, (c) Fe_3O_4-Au/GCE and (d) Fe_3O_4-Au-IL/GCE.

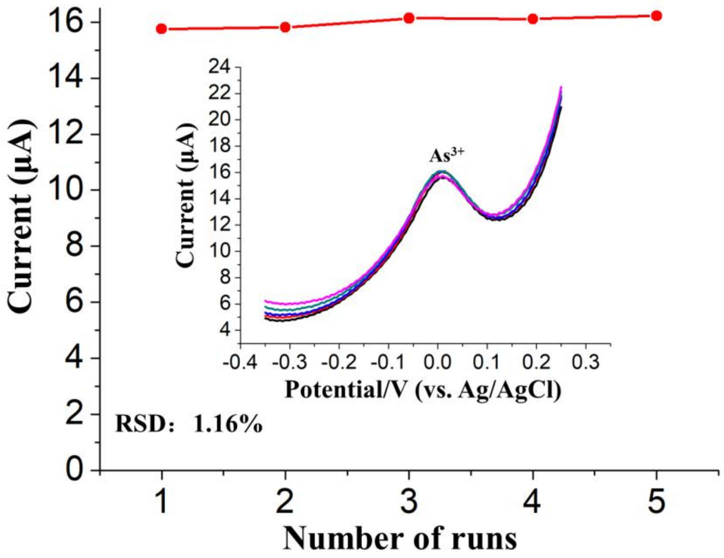

Figure S3. Five repetitive stripping current measurements of 60 μg/L As(III) using the Fe_3O_4-Au-IL/GCE in 0.2 M acetate buffer (pH 5.0). The insets are the data collected from every SWASV response for five runs.

References

1. Pungjunun, K.; Chaiyo, S.; Jantrahong, I.; Nantaphol, S.; Siangproh, W.; Chailapakul, O. Anodic stripping voltammetric determination of total arsenic using a gold nanoparticle-modified boron-doped diamond electrode on a paper-based device. *Microchim. Acta* **2018**, *185*, 324.
2. Lan, Y.; Luo, H.; Ren, X.; Wang, Y.; Liu, Y. Anodic stripping voltammetric determination of arsenic (III) using a glassy carbon electrode modified with gold-palladium bimetallic nanoparticles. *Microchim. Acta* **2012**, *178*, 153–161.
3. Chowdhury, A.; Ferdousi, S.; Islam, M.M.; Okajima, T.; Ohsaka, T. Arsenic detection by nanogold/conducting-polymer-modified glassy carbon electrodes. *J. Appl. Polym. Sci.* **2007**, *104*, 1306–1311.
4. Nunez-Bajo, E.; Blanco-López, M.C.; Costa-García, A.; Fernández-Abedul, M.T. Electrogeneration of Gold Nanoparticles on Porous-Carbon Paper-Based Electrodes and Application to Inorganic Arsenic Analysis in White Wines by Chronoamperometric Stripping. *Anal. Chem.* **2017**, *89*, 6415–6423.
5. Xie, Z.; Xu, J.; Xie, F.; Xiong, S. Electrochemical Detection of As (III) by a rGO/Fe3O4-modified Screen-Printed Carbon Electrode. *Anal. Sci.* **2016**, *32*, 1053–1058.
6. Majid, E.; Hrapovic, S.; Liu, Y.; Male, K.B.; Luong, J.H.T. Electrochemical determination of arsenite using a gold nanoparticle modified glassy carbon electrode and flow analysis. *Anal. Chem.* **2006**, *78*, 762–769.
7. Shin, S.-H.; Hong, H.-G. Anodic stripping voltammetric detection of arsenic (III) at platinum-iron (III) nanoparticle modified carbon nanotube on glassy carbon electrode. *Bull. Korean Chem. Soc.* **2010**, *31*, 3077–3083.
